# Evolving or Immutable - Phase I Solid Tumor Trials in the Era of Precision Oncology

**DOI:** 10.21203/rs.3.rs-4202155/v1

**Published:** 2024-04-29

**Authors:** Shannon S. Stockton, G. Dan Ayers, Cody Lee, Heather Laferriere, Satya Das, Jordan Berlin

**Affiliations:** Vanderbilt University Medical Center; Vanderbilt University Medical Center; Vanderbilt University Medical Center; Vanderbilt University; Cullgen, Inc; Vanderbilt University Medical Center

**Keywords:** Phase I, Clinical Trial, Precision Oncology

## Abstract

**Purpose:**

In the era of precision oncology (PO), systemic therapies for patients (pts) with solid tumors have shifted from chemotherapy (CT) to targeted therapy (TT) and immunotherapy (IO). This systematic survey describes features of trials enrolling between 2010–2020, focusing on inclusion criteria, type of dose escalation scheme (DES) utilized, and use of expansion cohorts (ECs).

**Methods:**

A literature search identified phase I studies in adults with solid tumors published January 1, 2000 – December 31, 2020 from 12 journals. We included only studies enrolling between 2010–2020 to better capture the PO era. Two reviewers abstracted data; a third established concordance.

**Results:**

Of 10,744 studies, 10,195 were non-topical or enrolled prior to 2010; 437 studies were included. The most common drug classes were TT (47.6%), IO (22%), and CT (6.9%). In studies which reported race, patients were predominantly white (61.7%) or Asian (25.7%), followed by black (6.5%) or other (6.1%). Heterogeneity was observed in the reporting and specification of study inclusion criteria. Only 40.1% of studies utilized ECs, and among the studies which used ECS, 46.6% were defined by genomic selection. Rule-based DES were used in 89% of trials; a 3+3 design was used in 80.5%. Of all drugs tested, 37.5% advanced to phase II, while 10.3% garnered regulatory licensure (for an indication tested in phase I).

**Conclusion:**

In the era of PO, TT and IO have emerged as the most studied agents in phase I trials. Rule-based DES, which are more relevant for escalating CT, are still chiefly utilized.

## Introduction

Phase I oncology trials represent the initial effort to test novel therapeutics in patients with advanced malignancies. The primary goal is to establish safety of new drugs and define recommended phase 2 doses (RP2Ds) for subsequent clinical trials^1^. A key secondary goal is to identify signals of anti-tumor activity in patients with certain tumor types who may derive the most benefit from the systemic therapy tested in the study. As we have entered the era of precision oncology (PO)^2^, the nature of systemic therapies for patients with solid tumors has fundamentally changed, from chemotherapy to targeted therapy (TT) and immunotherapy (IO). While PO has altered the conduct of drug development for patients with certain solid tumors (e.g., tissue agnostic and accelerated approvals), no study, to the best of our knowledge, has defined phase I trials in this era. In this systematic survey, we describe the characteristics of phase I solid tumor trials, that began enrollment between 2010–2020, focusing on features such as type of dose escalation scheme (DES) utilized, use and selection of expansion cohorts, participant race, study inclusion criteria, and nature of adverse events (AEs) (grade ¾ AEs and dose limiting toxicities (DLTs)) experienced by patients. We also interrogated the trial features which may predict whether a therapeutic tested in phase I is carried forward to phase II testing or garners regulatory licensure, to shed light on how we may be able to optimize future early-phase oncology trial design.

## Methods

### Search Methodology

A literature search was performed by a biomedical librarian to identify phase I studies of solid tumors published between January 1, 2000 – December 31, 2020 in a selection of oncology journals (Annals of Oncology, British Journal of Cancer, Cancer Discovery, Clinical Cancer Research, Investigational New Drugs, JAMA Oncology, Journal of Clinical Oncology, Lancet, Lancet Oncology, Molecular Cancer Therapeutics, The New England Journal of Medicine, and The Oncologist). PubMed (NCBI), EMBASE (OvidSP), Cumulative Index of Nursing and Allied Health Literature (CINAHL) (EBSCOhost), Web of Science Core Collection (Clarivate), Cochrane Database of Systematic Reviews (Wiley), and Cochrane CENTRAL Register of Controlled Trials (Wiley) were searched in March 2021. The search strategy consisted of a combination of keywords and database-specific subject headings and is described in detail in the supplementary material.

### Study Selection

We defined inclusion and exclusion criteria a priori. We included phase I studies in patients with solid tumors that began enrollment between 2010–2020. Although the initial search included studies conducted between 2000–2020, this analysis was restricted to studies enrolling between 2010–2020 to be more representative of study designs utilized in the era of PO. Studies were included if they were identified as a phase I trial or represented expansion cohorts (ECs) of a previously published phase I trial. Only studies in adults (age ≥ 18) and involving patients with unresectable or metastatic solid tumors were included. We included trials with both single and multiple agents, as well as multiple modalities, so long as the systemic therapy being tested was the one being escalated. We excluded studies which only involved intra-tumoral or intra-dermal therapies. We also excluded trials which were supportive care focused, non-interventional or included only non-human subjects. Two reviewers (SS and CL) evaluated the titles and abstracts of publications identified by the search strategy. Publications thought to be potentially relevant were retrieved in full. The reviewers then assessed full publications for eligibility; reviewers were not blinded to study authors or outcomes. When it was not clear whether a study met inclusion or exclusion criteria, final inclusion was determined by a third reviewer (SD).

### Data Abstraction

Data Abstraction was performed by SS and CL. Study information was obtained from the published manuscript and clinicaltrials.gov, not study protocols. To assess concordance between the abstraction techniques of both reviewers, a third reviewer (SD) reviewed 20 studies (randomly selected) which were initially abstracted by both of the two primary reviewers. A concordance rate of 98.7% was established between the techniques of SS and CL. Details of data abstraction are described in the supplementary material.

### Objectives

The primary aim of the analysis was to estimate the proportion of phase I trials that used rule-based DES. Rule-based DES include the 3+3 design or its variations (e.g., accelerated titration, Rolling 6). Secondary aims included qualitatively describing the ECs included in studies (e.g., genomic inclusion, sample size justification, tumor types included, endpoints), the listed objectives of studies, the mechanisms of action and administration of tested therapeutics, reported inclusion criteria, study demographics, the characteristics of AEs experienced by patients and characteristics of included studies. Additionally, this analysis sought to characterize trial features (supplementary material) associated with whether the study agent was further assessed in a phase II trial and whether the study agent eventually obtained regulatory licensure for the indication investigated in phase I testing.

## Results

**Figure 1:**
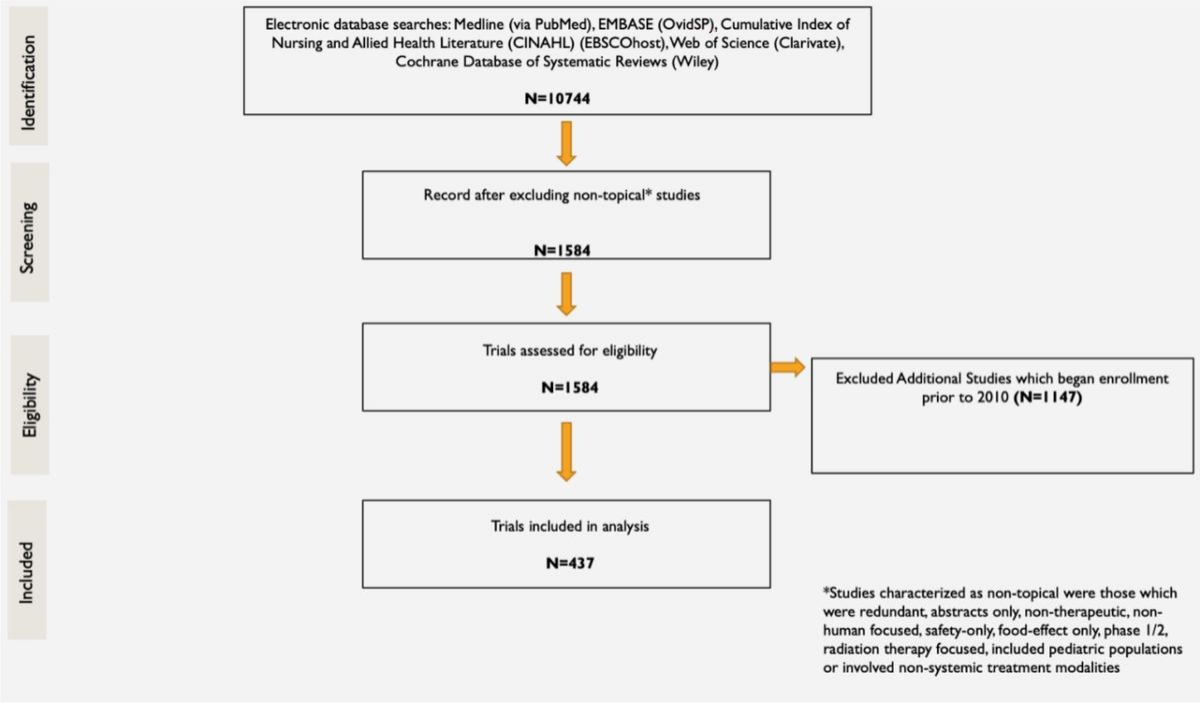
Study inclusion schematic

The initial literature search identified 10,744 studies that enrolled patients between 2000 and 2020. After excluding non-topical studies, 1,584 studies were assessed for eligibility. An additional 1,147 studies that enrolled between 2010 and 2020 were excluded (Figure 1).

Of the included 437 Phase I studies, 353 studies included a dose-escalation component. In the studies with dose escalation, rule-based DES were most utilized (89% of studies) whereas model-based DES were used less frequently (11% of studies). Among studies utilizing rule-based DES, the most common dose-escalation design (80.5%) was 3+3 (Table 1, Figure 3). Less frequent dose-escalation designs, including model-based schemes (mTPI, TITE-CRM, Bayesian CRM, modified CRM and BOIN), were represented in the “other category” (19.5% of included studies).

TTs represented the most common drug class tested (47.6%), while IO (22%) and “other” agents (20.1%) constituted the next most frequent therapeutic classes tested. Examples of drugs represented in the “other” category include antibody-drug conjugates and monoclonal antibodies against non-immune, non-antiangiogenic targets. Chemotherapeutic (CT) agents (6.9% of studies) and DNA damage repair inhibitors (3.4%) were utilized less frequently. The majority of tested agents were delivered intravenously (IV) (42.3%) whereas agents were orally administered in 30.7% of studies (Table 1). In total, 51.7% of participants and 48.3% of patients from all studies were categorized by gender as male and female, respectively. Out of 437 studies, 213 (48.7%) did not report the race of study participants. When accounting for all patients from studies which reported race, 61.7% were identified as white, 25.7% were identified as Asian, 6.5% were identified as black, and 6.1% were identified as “other” (Table 2). Examples of “other” race included Native American, multiracial, and Hispanic or Latino. Median lines of prior therapy for all study patients was 3 (IQR 2–4); median lines of prior therapy for patients in ECs was 2 (IQR 1–3). In the 429 studies that reported performance status (PS), 71.8% of patients were classified as ECOG PS 1, while 28.2% of patients were classified as ECOG 2.

With regards to inclusion criteria, the median creatinine clearance cutoff was ≥50 mL/min. Creatinine clearance cutoff was stated as an inclusion criterion but not specified in 41.6% of the total 437 studies. The median hemoglobin cutoff was ≥9 g/dL, and the hemoglobin cutoff was not specified in 42.6% of studies. The median white blood cell cutoff was ≥2000 units/μL. White blood cell count was reported as an inclusion criterion but the count was not specified in 43% of studies, and white blood cell count was not an inclusion criterion in 46% of studies. The median neutrophil count cutoff was ≥1500 units/μL, and 42.1% of studies reported neutrophil count as an inclusion criterion but did not specify the cutoff value. The median platelet count cutoff was ≥100,000 units/μL, and 41.9% of studies reported platelet count as an inclusion criterion but did not specify the cutoff value. The median albumin cutoff was ≥3 g/dL, and 93.1% of studies did not report albumin as an inclusion criterion. The median transaminase cutoff was ALT/AST ≤ 3, and 43.2% of studies did not specify the transaminase cutoff for inclusion. The median total bilirubin cutoff was bilirubin ≤ 2 times the upper limit of normal, and 43.2% of studies did not specify the bilirubin cutoff for inclusion (Table 2).

The most common primary objective of studies was to define dose-limiting toxicities (DLTs) and pharmacokinetics (PK; 74.7%). Additional primary objectives included “other” (24%) and objective response rate (ORR; 1.4%),The “other” category included objectives such as characterizing pharmacodynamics (PD), determining maximum tolerated dose and/or recommended phase 2 dose, and “not relevant” in the case of expansion-cohort only/Phase Ib studies (Table 1). Three studies did not report primary objective clearly. DLTs and Grade 3/4 Adverse Events were assessed and reported in Supplementary Table 1.

ECs were utilized in 39.8% of the evaluated studies and were defined genomically in 46.6% of studies which included them (Table 1); sample size justification for the ECs was not provided in 71.3% of studies which utilized them. In studies that did justify EC sample size, target ORR was most often cited (20.7%). The number of tumor types included in ECs varied significantly, from 1 to 10 tumor types per study. The most common primary endpoint of ECs was to determine PK and DLTs (57.1%), followed by ORR determination (37.6%). Out of the 174 studies utilizing ECs, 140 of them (80.5%) were industry-funded.

Most studies were multi-center (73.5% of studies reported specific number of centers). Studies were predominantly conducted in North America (46.5%); international trials occurred less commonly (25.6%) (Table 1). Industry sponsorship was the most common funding mechanism for studies (71%) while 11.3% of studies were funded by national cancer institutes (NCI). “Other” sponsorship was used in 17.7% of studies and included non-NCI, non-industry funding, such as private grants, or combination funding, such as industry and NCI sponsorship.

Therapeutics tested in phase I were further tested in phase 2 trials in 37.5% of studies, while 10.3% of these agents ultimately received regulatory approval for an indication included in phase I testing.

**Figure 2:**
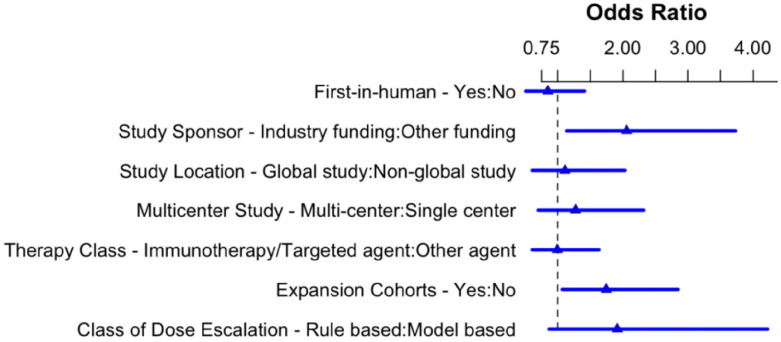
Multi-variate analysis of factors associated with subsequent phase 2 testing

**Figure 3:**
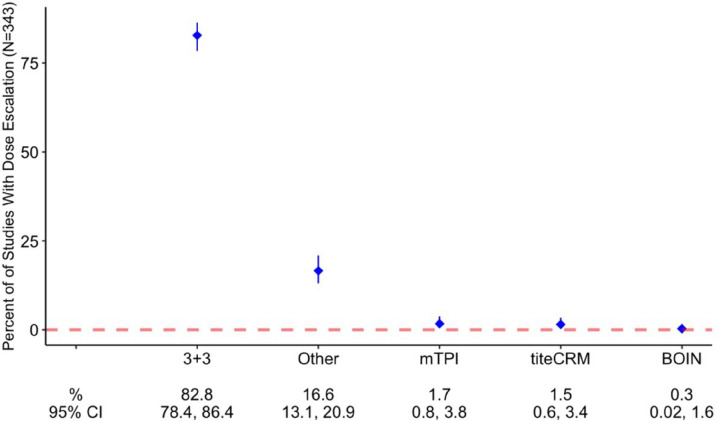
Dose-Escalation Designs

On univariate analysis, factors associated with whether a therapeutic subsequently underwent phase 2 testing included industry funding, international conduct, multicenter involvement, and use of expansion cohorts (Pearson chi-square p<0.01). On multivariate (logistic regression) analysis, only industry funding [OR 2.08, 95% CI 1.15–3.78] and utilization of expansion cohorts [OR 1.71, 95% CI 1.05–2.80] remained significantly associated with subsequent phase 2 testing (Figure 2). Regulatory approval for a therapeutic was linked to industry funding, international conduct, multicenter involvement, and use of ECs in univariate analysis (p<0.01). In multivariate analysis, none of these variables maintained statistical significance for an association with subsequent regulatory approval (Supplementary Figure 1).

## Discussion

Rule-based DES dominate phase I oncology trials to date, with 3+3 representing the primary dose escalation design^3^. While there are advantages to utilizing the 3+3 design, namely ease of use and safety (e.g., identification of clinically relevant toxicity and low rates of treatment related death)^4^, there are also important limitations. One of the primary limitations of the 3+3 design is that it is designed for CT with a monotonic relationship between dose, toxicity and anticipated response^5^. Thus, defining maximum tolerated dose (MTD) of an agent is crucially important for identifying the recommended phase 2 doses (RP2D) for subsequent drug development. Novel therapeutics such as TT and IO possess different intrinsic relationships between dose, toxicity and efficacy; the RP2D of an agent may be the biologically effective dose (BED) rather than MTD^6^. Another primary limitation of the 3+3 design is that it requires a prespecified attribution of toxicity for each dose level. This is easier to define for CT based upon the anticipated toxicity from a CT class (e.g., neuropathy and hematologic toxicity from platinum agent), but may be more unpredictable when attempting to define anticipated toxicities from TT or IO. In our analysis, TT (47.6%) and IO (22%) were more commonly utilized than CT (6.9%). Newer DES, which rely on Bayesian principles, allow emerging toxicity at a particular dose level to inform optimal toxicity thresholds. Our analysis reflects some shift in DES, with rule-based DES being utilized in 89% of studies (80.5% which used the 3+3 design), compared to 96.7% of the time in phase I oncology studies published between 1991–2006^1^,^7^. However, there is still room for improvement, and healthcare authorities have recognized this need for reform. In 2021 the FDA Oncology Center of Excellence announced Project Optimus, an initiative to reform dose selection and optimization for novel therapeutic agents (FDA Project Optimus) in the era of PO^8^. This initiative serves as an acknowledgement by the FDA that in the modern therapeutic landscape, establishing maximum tolerated dose by rule-based DES such as 3+3 may no longer be optimal to define doses for further testing. Project Optimus is ongoing and encourages strategies such as trial designs which include model-based DES and comparison of multiple doses (e.g., parallel dose-response). Nearly half of studies included in this analysis did not report the race of participants (213/437 studies, 48.7%). It is challenging to draw meaningful conclusions about racial representation when a large proportion of trials are not reporting this data. Additionally, the absence of patient race identification precludes gathering data about possible differences in dosing for different subpopulations. Based on the predominance of white patients in these studies, it is unclear whether dosing information can be generalized to other faces. While the reported gender of study participants was fairly balanced (51.7% male, 48.3% female), the majority of patients on studies in this analysis identified as white (61.7%), and only 6.5% of patients identified as black. The “other” category, which captured Hispanic or Latino patients, only represented 6.1% of patients. The greatest proportion of studies were based in North America (46.5%), and based on United States census data from July 2023, 75.5% of Americans identify as white, 13.6% of Americans identify as black, and 19.1% of Americans identify as Hispanic or Latino^9^. Based on our analysis, minority populations are still being underrepresented in clinical trials. Since 25.6% of analyzed studies were conducted on 2 or more continents, we would further expect more diversity in the patient population. However, no trials were conducted on the African continent and few trials in South America, somewhat further restricting diversity.

Additionally, despite surveying a heavily pretreated patient population with a median of 3 prior lines of therapy, most studies (71.8%) required an ECOG performance status (PS) of 1. The functional status of the real-world population of patients who have received multiple therapies may not reflect the high-functioning population able to enter a trial, limiting generalizability of results. Notably, the subjective nature of PS may undermine its ability to accurately predict which patients are suitable for a particular therapy. Nonetheless, the ASCO-Friends of Cancer Research Performance Status Work Group conducted a simulation study which demonstrated that including relatively small numbers of ECOG 2 participants had only modest effects on treatment hazard ratio and study power, and expanding eligibility may lead to shorter trial duration as a result of faster accrual. The working group has set forth recommendations to consider expanding PS eligibility^10^. This is of import in the era of precision oncology, as novel agents with differing toxicities from traditional cytotoxic chemotherapy may be more tolerable in frailer patients. A more precise definitionof PS may also be helpful, such as Karnofsky PS which has more categories than the more commonly used, ECOG PS.

Among all lab value inclusion criteria analyzed except albumin, at least 40% of inclusion cutoff values were not clearly specified (Table 2). Clearly identifying clinical characteristics required for entering a study is critical in accurately defining the study population eligible for a particular therapy. In the case of albumin, 93.1% of studies did not state its use as an inclusion criterion. Prior work evaluating mortality rates of patients in phase I trials has suggested that lower albumin levels (3.3 g/dL) are associated with higher rates of death within 90 days, independent of ECOG PS ^11^. Lower albumin levels have been associated with decreased survival rates in numerous studies^12–15^. Greater utilization of albumin as a study inclusion criterion may aid in appropriately excluding patients at high risk for clinical deterioration. As the aim of phase I oncology trials has expanded beyond safety to signal-finding, ECs have been increasingly utilized^15^. Use of ECs and size of ECs (> 20 patients) in these trials has been associated with drug success in later lines of development^16^. Our data lends further credence to this idea, as the use of ECs was significantly associated with progression to phase 2 testing in multi-variate analysis. However, we cannot account for the fact that planned ECs may have been eliminated when the drugs showed little sign of activity in the escalation phase, thus skewing our results. In this analysis, industry studies were much more likely to include ECs than non-industry funded studies (p<0.01). Given the strong association of EC use in phase I trials with subsequent phase II testing, it is plausible that differential EC utilization was the underlying reason why phase I industry trials appeared more likely to lead to phase II testing than non-industry trials. Our analysis demonstrates the continued increase in utilization of ECs, with 40% of all studies including such cohorts (24% in prior analysis^15^). Among studies utilizing ECs, the primary objective was listed explicitly in 76.4% of cases. This represents an improvement from prior analyses in which primary objective definition was not stated clearly. Despite the increasing utilization of ECs, sample size justification for the cohorts was not provided in most trials (71.1%). In the studies which justified EC size, target ORR threshold was utilized 20.3% of trials. FDA guidance for EC use in phase I trials suggests clear sample size justification in the statistical analysis plan may facilitate more seamless development for a drug based on more concrete signals of anti-tumor activity^18^. This is a glaring area of weakness in current phase I oncology trial design which can be remedied quite easily in our estimation. Given the increasing use of biomarker selection for ECs (46.6% in our analysis), target response rate thresholds should be easier to estimate. Simple response threshold-based sample size justification (e.g., Simon’s two-stage design) should become a mainstay of statistical analysis plans for studies involving ECs, given this approach will optimize signal-finding in phase I oncology trials.

### Limitations

We note the following limitations in our analysis. First, hematologic malignancies were not represented in this analysis given the inherent differences between most drugs used in hematologic versus solid tumor malignancies. It is an established practice to separate malignancies in this fashion in previously published studies. Second, we acknowledge the possibility of discrepancies in methodology between the two data abstractors. This was mitigated by an independent third reviewer who established 98.7% concordance between the work of the primary abstractors. Third, we relied on trial information presented in publications and published on clinicaltrials.gov instead of study protocols, given the variability in access to full-length study protocols. Since data was only considered from published manuscripts, there was inherent publication bias (towards positive studies) in the analysis. To mitigate this risk, we included a diverse array (with impact factors ranging from low to high) of journals with a track record of publishing phase I studies. Fourth, we acknowledge by excluding studies with multiple dose cohorts or multiple drug combinations reported separately, and phase I/II studies, we may have underrepresented more recent novel therapeutics by excluding these types of studies. However, we restricted our analysis to phase I only trials to most purely assess the evolving landscape of these trials. The number of studies included in the analysis (N=437) reduces the concern about the generalizability of our conclusions.

## Conclusion

Since 2010, TT and IO agents are being studied more commonly than CT in phase I trials. Despite this trend, rule-based DES, which are more relevant for escalating CT, are still most frequently utilized. Beyond increasing the use of model-based DES, expanding EC use and defining selection of ECs could enhance the development of therapeutics currently being tested in phase I solid tumor trials.

## Figures and Tables

**Table 1:** Characteristics of Included Trials

Dose Escalation Scheme (N=353)	
Rule	314 (89%)
Model	39 (11%)
**Dose Escalation Design (N=353)**	
3+3	284 (80.5%)
Other	57 (19.5%)
mTPI	6 (1.7%)
TITE-CRM	5 (1.4%)
BOIN	1 (0.3%)
**Most Common Drug Classes (N=437)**	
Targeted Therapy	208 (47.6%)
Immunotherapy	96 (22%)
Other	88 (20.1%)
Chemotherapy	30 (6.9%)
DNA Damage Repair Inhibitor	15 (3.4%)
**Administration Route (N=437)**	
IV	185 (42.3%)
Oral	134 (30.7%)
IV+Oral	71 (16.2%)
Other	45 (10.3%)
Intra-peritoneal	2 (0.5%)
**Primary Endpoint (N=434)**	
DLT/PK	324 (74.7%)
Other	104 (24%)
ORR	6 (1.4%)
**Use of Expansion Cohorts (ECs; N=437)**	
Yes	174 (39.8%)
No	263 (60.2%)
**EC Genomic Selection (N=174)**	
Yes	81 (46.6%)
No	93 (53.4%)
**EC Sample Size Justification (N=174)**	
Not Listed	124 (71.3%)
Target Objective Response Rate	36 (20.7%)
Other	11 (6.3%)
Pharmacokinetics	3 (1.7%)
**Primary Endpoint of ECs (N=174)**	
DLT/PK	76 (43.7%)
Not Listed	41 (23.6%)
ORR	50 (28.7%)
Other	7 (4.0%)
**Number of Centers (N=437)**	
Multiple	313 (71.6%)
Single	113 (25.9%)
Not Listed	11 (6.4%)
**Study Location (N=437)**	
North America	203 (46.5%)
Global	112 (25.6%)
Asia	58 (13.3%)
Europe	55 (12.6%)
Other	9 (2.1%)
**Study Sponsorship (N=437)**	
Industry	308 (71%)
Other	77 (17.7%)
NCI	49 (11.3%)
Not Listed	3 (0.7%)

**Table 2:** Demographics and Inclusion Criteria

Sex (total number of patients in all studies)	
Male	9229 (51.7%)
Female	8632 (48.3%)
**Race (total number of patients in all studies)**	
White	4267 (61.7%)
Asian	1776 (25.7%)
Black	447 (6.5%)
Other	424 (6.1%)
**Race Not Reported (n=437 studies)**	213/437 studies (48.7%)
**Median Lines of Prior Therapy, Expansion Cohorts[n (IQR)]**	2 (1–3)
**Performance Status (n=429 studies)**	
ECOG 1	71.8%
ECOG 2	28.2%
**Inclusion Criteria (n=437 studies)**	
**Creatinine Clearance**	
Median Creatinine Clearance (mL/min)	≥50
Creatinine Clearance Not Specified	182/437 studies (41.6%)
Creatinine Clearance Not Included	33/437 studies (7.6%)
**Hemoglobin**	
Median Hemoglobin (g/dL)	≥9
Hemoglobin Not Specified	186/437 studies (42.6%)
Hemoglobin Not Included	70/437 studies (16.0%)
**White Blood Cell Count**	
Median White Blood Cell Count (units/µL)	≥2000
White Blood Cell Count Not Specified	188/437 studies (43.0%)
White Blood Cell Count Not Included	201/437 studies (46.0%)
**Neutrophil Count**	
Median Neutrophil Count (units/µL)	≥1500
Neutrophil Count Not Specified	184/437 studies (42.1%)
Neutrophil Count Not Included	35/437 studies (8.0%)
**Platelet Count**	
Median Platelet Count (units/µL)	≥100,000
Platelet Count Not Specified	183/437 studies (41.9%)
Platelet Count Not Included	34/437 studies (7.8%)
**Albumin**	
Median Albumin (g/dL)	≥3
Albumin Not Specified	10/437 studies (2.3%)
Albumin Not Included	407/437 studies (93.1%)
**Transaminases**	
Median Transaminases (units/L)	ALT/AST ≤ 3
Transaminases Not Specified	189/437 studies (43.2%)
Transaminases Not Included	33/427 studies (7.6%)
**Total Bilirubin**	
Median Total Bilirubin (mg/dL)	Bilirubin ≤ 2 times ULN
Total Bilirubin Not Specified	189/437 studies (43.2%)
Total Bilirubin Not Included	33/437 studies (7.6%)
